# A multilevel study of the determinants of area-level inequalities in colorectal cancer survival

**DOI:** 10.1186/1471-2407-10-24

**Published:** 2010-01-28

**Authors:** Peter D Baade, Gavin Turrell, Joanne F Aitken

**Affiliations:** 1 Viertel Centre for Research in Cancer Control, Cancer Council Queensland, PO Box 201, Spring Hill QLD 4004, Australia; 2 School of Public Health, Queensland University of Technology, Herston Road, Kelvin Grove 4059, Australia; 3 School of Population Health, University of Queensland, Herston Road, Herston 4061, Australia

## Abstract

**Background:**

In Australia, associations between geographic remoteness, socioeconomic disadvantage, and colorectal cancer (CRC) survival show that survival rates are lowest among residents of geographically remote regions and those living in disadvantaged areas. At present we know very little about the reasons for these inequalities, hence our capacity to intervene to reduce the inequalities is limited.

**Methods/Design:**

This study, the first of its type in Australia, examines the association between CRC survival and key area- and individual-level factors. Specifically, we will use a multilevel framework to investigate the possible determinants of area- and individual-level inequalities in CRC survival and quantify the relative contribution of geographic remoteness, socioeconomic and demographic factors, disease stage, and access to diagnostic and treatment services, to these inequalities. The multilevel analysis will be based on survival data relating to people diagnosed with CRC in Queensland between 1996 and 2005 (n = 22,723) from the Queensland Cancer Registry (QCR), area-level data from other data custodians such as the Australian Bureau of Statistics, and individual-level data from the QCR (including extracting stage from pathology records) and Queensland Hospitals. For a subset of this period (2003 and 2004) we will utilise more detailed, individual-level data (n = 1,966) covering a greater range of risk factors from a concurrent research study. Geo-coding and spatial technology will be used to calculate road travel distances from patients' residence to treatment centres. The analyses will be conducted using a multilevel Cox proportional hazards model with Level 1 comprising individual-level factors (e.g. occupation) and level 2 area-level indicators of remoteness and area socioeconomic disadvantage.

**Discussion:**

This study focuses on the health inequalities for rural and disadvantaged populations that have often been documented but poorly understood, hence limiting our capacity to intervene. This study utilises and develops emerging statistical and spatial technologies that can then be applied to other cancers and health outcomes. The findings of this study will have direct implications for the targeting and resourcing of cancer control programs designed to reduce the burden of colorectal cancer, and for the provision of diagnostic and treatment services.

## Background

Colorectal cancer is the second most commonly diagnosed invasive cancer in Australia, with 13,076 people being diagnosed in 2005 (representing 13.0% of all new cancer diagnoses) and 4,168 dying from the disease (10.7% of all cancer deaths and 3.2% of all deaths) [1]. Australian incidence rates for colorectal cancer are among the highest in the world [2] and it is the third highest cancer contributor to health care expenditure in Australia (estimated at about $235 million annually) [3]. Colorectal cancer is associated with considerable physical and psychological morbidity [4], due in part to the side-effects of surgery, radiation, and systemic therapies. While incidence rates are generally steady, numbers of new colorectal cancer diagnoses have been increasing with population growth [1].

### Rural variations

Significant geographical variation in colorectal cancer survival has been reported across Australia, with lower survival estimates for people diagnosed outside major cities [5-7], even when adjusted for spread of disease [6,8]. Residents of rural areas of Queensland diagnosed with colorectal cancer have 5-year survival up to 14% lower compared with those in the highly urbanised south-east corner [5]. Similar inequalities have been reported for other Australian states: when adjusted for stage, there was up to a 30% increased excess mortality risk from colon cancer within 5 years of diagnosis for residents of more rural, less accessible areas in New South Wales compared to highly accessible areas [6].

The reasons for rural inequalities in colorectal cancer survival are complex and multi-faceted [9] and include population and family age structure, socioeconomic status, diet, ethnicity and Indigenous status, environmental, industry and occupational exposures to carcinogens, access to cancer screening and diagnostic services, access to cancer treatment services, and level of co-morbidities with other diseases such as cardiovascular disease and diabetes [10].

Since cancer stage is the most strongly predictive of all clinical prognostic factors, it may be the most intuitive explanation for the rural inequalities in colorectal cancer survival. Differences in stage have been shown to be a key explanation for international differences in colorectal cancer survival [11]. In Australia however rural inequalities in colorectal cancer survival have remained even after adjusting for spread of disease [6]. While it may be logical to suggest that rural people are diagnosed with more advanced cancers, a US study found that the opposite was true, in that urban patients were more likely to present with later stage colorectal cancer than rural patients [12]. So although disease stage could potentially explain a portion of the observed rural inequalities in colorectal cancer survival, it is unlikely to be the sole explanation.

Indigenous Australians comprise a larger proportion of rural and remote areas than urban areas [13]. Although the incidence of colorectal cancer among Indigenous Australians has been reported to be substantially lower than non-Indigenous Australians [14], comparisons of the mortality:incidence ratios suggest that once diagnosed with colorectal cancer, Indigenous Australians have poorer survival [14].

Location of and access to health services have been recognised as important contributors to morbidity and mortality in Australia. Health care services in Australia are becoming increasingly centralised [15], with three quarters of all radiation therapy facilities in Australia in 2002 being in capital cities [16]. There is merit in this centralisation of oncology services; evidence suggests that the best outcomes are obtained when patients are treated by practitioners and institutions with high caseloads [17]. However this centralisation has implications for rural cancer patients' access to diagnostic and treatment services, and the increased distances rural patients need to travel to use those services that may a disincentive to undertake or complete treatment regimens. This is particularly relevant when the treatment involves a series of specific regimes, such as radiotherapy, that may involve prolonged absence from home resulting in disruptions to normal life and financial hardship [18].

Previous studies have shown that cancer patients in rural and remote areas of Australia have reduced access to cancer care services. These include fewer radical prostatectomies for prostate cancer [19]; lower proportions of breast-conserving surgery [20]; and a lower likelihood of completing radiotherapy treatment for rectal cancer [21]. Despite national increases in the number of general practitioners (GPs) per capita, numbers of GPs have decreased in rural and remote areas [22]. Internationally, studies have demonstrated a direct association between distance to cancer treatment services and patients' use of that treatment, with patients less likely to access specialist treatment when longer distances are involved [23-25].

### Socio-economic variations

There continues to be a strong association between socio-economic status (SES) and cancer survival internationally, with colorectal cancer survival rates typically 25%-35% lower among the most deprived populations compared to the most affluent, even after adjustment for spread of disease [26]. Reports from Australia [5,27] suggest similar (but not significant) trends in colorectal cancer survival by SES.

As is the case for rural inequalities, cancer stage is one possible explanation for the socioeconomic inequalities in colorectal cancer survival. International studies have demonstrated that socio-economic inequalities reduced, but still remained after adjustment for stage at diagnosis [26]. While advanced stage at colorectal cancer diagnosis is generally more common among deprived people, it is not always the case [26]. Therefore although disease stage potentially explains some of the socioeconomic survival inequalities in colorectal cancer (as with rural inequalities), it can't be assumed it will explain all, or even most, of the differences.

While physical inactivity and obesity have been shown to increase the risk of developing colorectal cancer, relatively little is known about the risk factors for disease progression and survival. However there is an emerging body of evidence suggesting that higher intakes of Western diets (including high amounts of red meat and fat) [28], reduced physical activity [29], increased BMI [30], smoking [31] and the presence of co-morbid conditions [32] are associated with reduced survival for colorectal cancer patients. If the prevalence of these risk factors differs according to SES, as has been suggested for physical activity [33], then this could be one explanation for the socioeconomic inequalities in colorectal cancer survival.

There is increasing international evidence of colorectal cancer patients in different socioeconomic groups being given different treatment [26], with affluent colorectal cancer patients in England being more likely to receive surgery than disadvantaged patients [26]. In the United States, people living in poorer areas and those without private health insurance were less likely to receive recommended adjuvant chemotherapy or radiotherapy for stage II or III colon cancer [26]. The few Australian studies [34] on this topic have suggested similar patterns of reduced access to hospital services by colorectal cancer patients with lower socioeconomic status.

### Limitations of previous studies

Current classifications of rurality [35] are typically based on access to services. However they may be limited in their specific application to cancer outcomes because they do not place particular importance on access to cancer-specific services. In Queensland (Australia) the Area Remoteness Index of Australia [35] can classify some areas as being less accessible than others even though they are much closer to specialised cancer treatment and tertiary hospital services. Due to the multifaceted nature of rurality, it is important to measure the independent effects of factors such as access to services and distance to radiation treatment facilities, as well as the Indigenous component. The sizeable Indigenous populations in remote areas [13] and their poorer survival from cancer compared to other Australians [14] highlights the importance of separating the remoteness and Indigenous effects.

In the same way, broad classifications are typically are used to define socioeconomic status and assess socioeconomic inequalities. People with low incomes may live in very affluent areas, while the low socioeconomic areas may include people with high individual incomes. This lack of homogeneity with the SES areas may tend to dilute any observed association between cancer survival and socio-economic status [36]. As with rurality, the concept of socio-economic status is multi-faceted, thus relying on broad SES categories removes the opportunity to investigate the separate components of socioeconomic status, such as the independent effects of income or education.

This study will use a multilevel approach to investigate the possible determinants of area- and individual-level inequalities in colorectal cancer survival: and quantify the relative contribution of geographic remoteness, socioeconomic and demographic factors, disease stage, and access to diagnostic and treatment services, to these inequalities. It will thus overcome many of the methodological and interpretive problems of standard ecologic studies. By allowing for the partitioning and modelling of complex sources of area- and individual-level variation we will be able to determine whether areas have an impact on colorectal cancer survival independently of the characteristics of people who live in the areas. Without this information it is likely that our ability to address the inequalities in colorectal cancer survival will continue to be compromised.

## Study Aims

1. To quantify the extent of area-level variation in colorectal cancer survival among patients diagnosed in Queensland between 1996 and 2005

2. To assess the extent to which area-level variation in colorectal cancer survival is due to individual-level factors (i.e. patient characteristics, disease stage, co-morbidity, and access to health care and treatment services); and to examine the independent contribution of these individual-level factors to colorectal cancer survival.

3. To assess the extent to which area-level variation in colorectal cancer survival is associated with geographic remoteness and area socioeconomic disadvantage, after adjustment for individual-level factors.

4. To determine whether the relationship between individual-level factors and colorectal cancer survival differs by extent of geographic remoteness and area socioeconomic disadvantage.

## Methods/Design

### Funding and Support

This project was awarded funding by the (Australia) National Health and Medical Research Council (ID: 561700). Cancer Council Queensland provided additional funding for the maintenance of the GIS software.

### Ethical Clearance

This project was awarded ethical clearance by the Behavioural and Social Sciences Ethical Review Committee at the University of Queensland (Project No. 2008002041). Additional clearance to access confidential health information was obtained from the Research Ethics and Governance Unit, Queensland Health.

### Study Design

This study is a cross-sectional multi-level study (1996-2007)

### Setting

This study is being conducted using data relating to people diagnosed with colorectal cancer in Queensland (Australia) between 1996 and 2007 (inclusive). Queensland is the second largest state by area in Australia, and the third largest by population (4.4 million in 2009). It has the most decentralised population of any of the Australian states [37].

### Study areas

Three spatial units comprising the Australian Standard Geographical Classification (ASGC) will be used within Queensland - statistical divisions (SD), statistical subdivisions (SSD), and statistical local areas (SLA). These units cover Queensland without gaps or overlap. The SLAs, often based on the incorporated bodies of local governments who are responsible for service provision and infrastructure at the local and regional level, will be the primary focus for the area-level analysis. In 2006 there were 478 SLAs in Queensland with a median population of 5810 (range: 7 to 77523). The SLA is also used as the standard geographical area definition by most relevant data providers, in particular the QCR and Australian Bureau of Statistics. To account for SLA boundary changes over time we have applied a concordance developed and purchased from the Australian Bureau of Statistics to convert all SLA boundaries to the 2006 ASGC definition.

### Study participants

Details on all 22,723 colorectal cancer patients diagnosed in Queensland over the 10 year period between 1^st ^January 1996 and 31^st ^December 2005 were obtained from the Queensland Cancer Registry (QCR) via approved processes. Notification of cancer is a statutory requirement for all public and private hospitals, nursing homes and pathology services. Queensland pathology laboratories provide copies of pathology reports for cancer specimens to the QCR. The QCR also records details of SLA at diagnosis, as well as full address at last notification for geocoding purposes.

A separate longitudinal study of 1966 Queensland residents diagnosed with colorectal cancer between 2003 and 2004 while aged between 20 and 80 years has been conducted [38] by the Cancer Council Queensland, the cohort of which will be used for this study.

### Sample size and power calculations

Detailed power calculations require specific estimates of the variation in colorectal cancer survival between and within area units. Since the quantification of this variation is the primary goal of this study, and the number of records available to use are fixed, our retrospective power calculations (using *Optimum Design *software [39]) are based solely on the approximate number of clusters and records per cluster.

The average 5-year cause-specific survival for colorectal cancer patients in Queensland between 1996 and 2002 ranged from 56% for urban affluent to 50% in rural areas (unpublished data, Queensland Cancer Registry). There were 22,723 people diagnosed with colorectal cancer in Queensland between 1996 and 2005. This colorectal cancer cohort covers all 479 SLAs with between 1 and 416 (mean = 47, median = 28) patients in each SLA. Using a binomial approximation, and assuming 479 clusters with an average of 42 records per cluster and a baseline survival proportion of 50%, this gives 85% power at 0.05% significance to detect a difference in survival proportions of 2%.

The sub-study of 1,966 respondents to the longitudinal study between 2003 and 2004 covered 376 SLAs with between 1 and 46 (mean 5.2, median = 4) respondents in each SLA. By combining SLAs with similar rurality and socioeconomic characteristics, and so assuming 100 clusters with an average of 20 records per cluster and a baseline survival proportion of 50%, this gives 80% power at 0.05% significance to detect a difference in survival proportions of 6.5%.

## Data collection

### Area-level data

#### Location of cancer treatment centres

The caseload of CRC-related surgical procedures in Queensland public and private hospitals since the 1995/96 financial year will be obtained from the Queensland Hospital Admitted Patient Data Collection (QHAPDC), which contains information of all surgical episodes of care for patients admitted to Queensland public and private hospitals. Treating hospitals will be categorised into high volume and low volume based on the hospital-specific number of patients who had a colorectal cancer-related surgical procedure, a method demonstrated previously for breast cancer [40]. Additional details about the presence of multidisciplinary teams, chemotherapy and radiotherapy services in specific hospitals since 1996 will be obtained through relevant publications [16] and discussions with oncology clinicians and other health professionals. This will be supplemented by unstructured telephone surveys of oncology nurses and clinicians in Queensland hospitals, most of which have already been completed. Full street address information is available for all public and private hospitals.

#### Socioeconomic indicators

Information about education levels, household income, types of occupation, median mortgage and rental payments and other socioeconomic indicators for each SLA will be obtained from the census data files released by the Australian Bureau of Statistics for 1996, 2001 and 2006. We will also use the ABS SEIFA index of economic disadvantage, which is based on the percentage of people in the SLA with low income, low educational attainment or who are unemployed or employed in relatively unskilled occupations (among others). While acknowledging the weaknesses of this index [41], we will assess the impact this index has on measured socioeconomic inequalities compared to the more detailed information. Standard interpolation methods will be used to estimate these values in intervening years.

#### Life expectancy

The statistical modeling in this study utilizes cause-specific survival. An adjustment will be made for underlying mortality to account for general mortality differentials. Average life expectancy will be calculated from the unit record mortality file for Queensland from the Australian Bureau of Statistics that contains details of all-cause mortality by SLA.

#### Remoteness

Standard measures of remoteness classifications [35], and derived measures based on distances from major centres will be used, in addition to new SLA-based classifications based on distance to cancer treatment facilities.

### Individual level variables

#### Stage at diagnosis

The interpretation of differences in cancer survival estimates between population subgroups requires accurate information on cancer stage [11]. Since clinical stage is not routinely collected by population-based cancer registries in Australia, including the QCR, options include resource intensive medical chart reviews, or sourcing from pathology reports. Colorectal cancer is one of the most amenable to extracting stage from pathology reports, with the main limitation being a lack of information about metastasis [42]. Our recent study in Queensland, using data from a longitudinal study of colorectal cancer patients reported 80% agreement between stage extracted from pathology reports and stage obtained from clinical specialists [43]. This agreement increased to 95% when collapsing stage to A/B and C/D. We expect that approximately 85% of records will have sufficient information to assess stage, similar to that reported in the SEER registries (86%) [44] and in New South Wales (82%) [45]. Validity checks of test-retest reliability, inter-rater reliability, and agreement with clinical specialists [43]) will be conducted for a subset of the pathology records to maintain the accuracy of stage extraction.

#### Personal demographic information

The QCR collects information on date of diagnosis, place of usual residence at diagnosis, age group at diagnosis, sex, country of birth, marital status and occupation. Occupation is a mandatory data item, with 86% of colorectal cancer patients diagnosed in Queensland between 1996 and 2005 having a known occupation recorded, while 98% had a known marital status recorded (personal communication, Queensland Cancer Registry).

#### Indigenous status

The QCR collects data on the Indigenous status of people diagnosed with cancer in Queensland, and these data are available for the whole study period. Between 1996 and 2005 92% of colorectal cancer patients diagnosed in Queensland had a known Indigenous status recorded (personal communication, Queensland Cancer Registry). Although there is evidence of some under-identification, the Indigenous identifier in Queensland is considered to have good coverage [46].

#### Mortality

Deaths among the colorectal cancer cohort are routinely identified by the QCR by matching patient information to the Office of the Queensland Registrar of Births, Deaths and Marriages and to the National Death Index at the Australian Institute of Health and Welfare. Cancer registries in other states and territories also provide information on interstate deaths. People who were not known to have died are assumed to be still alive. Each member of the colorectal cancer study cohort will have at least two years of potential mortality follow-up to 31^st ^December 2007.

#### Location where surgical treatment received

By utilising a deterministic link between the QHAPDC and the QCR (based on treatment facility and hospital unit record number) we can identify at which specific hospital each colorectal cancer patient actually received surgical treatment. This process has already been successfully utilised for a patterns of care study [40].

#### Co-morbidities

Utilising the linkage between the QCR and QHAPDC described earlier, co-morbidities from each patient episode will be identified based on principal or other diagnosis codes (ICD-9-AM and ICD-10-AM classifications). While these diagnosis codes are limited to those conditions that specifically affected the inpatient's management in terms of treatment, diagnostic procedures or monitoring, they will provide information about important co-morbidites such as diabetes and cardiovascular disease.

#### Cancer treatment information

Information is available from the colorectal cancer longitudinal study [38] regarding the details of surgical treatment, chemotherapy and radiation therapy that the patients received, including geographical location details.

#### Detailed demographic and risk factor data

The colorectal cancer longitudinal study [38] provides information about the diagnostic process, quality of life, behavioural risk factors such as physical activity and body mass index, along with detailed demographic information. Survival information will also be available.

### Distance calculations

All address details for colorectal cancer patients, treatment facilities and diagnostic services will be geocoded using MapInfo MapMaker^®^, a commercial package designed to clean and convert address information into latitude and longitude coordinates. When full street address information is available, this will be used for geo-coding purposes. When full street address information is not available the centroid of the SLA will be used. In 1998 approximately 84% of colorectal cancer patients had full street address information, which increased to 93% in 2004 (personal communication, Queensland Cancer Registry).

Distances between patients, treatment facilities and other centres will be calculated directly from the geo-coded latitude and longitude points. Initially distance will be measured in terms of straight line "as the crow flies", as have other international studies [23-25]. However since the method can underestimate travel times [24] we will also calculate distance according to road travel distance and road travelling time. These road distances will be calculated using MapInfo Professional^®^, combined with street network analysis and display tools. A custom application has been developed by MapInfo to calculate the closest road travel distance between one location (such as patient's residence) and multiple locations (such as various treatment facilities).

## Analyses

### Multilevel perspective

Previous Australian studies examining area variation in colorectal cancer survival have used an aggregate ecologic design [5,27]. The conceptual and statistical problems with these designs have been well documented [47]. Ecologic studies that use data aggregated to a single geographic scale cannot provide a quantification of the variation between areas in terms of their survival profiles, and then, more importantly, indicate whether the variation is likely due to the clustering of individuals (i.e. a composition effect) or the environmental characteristics of the areas (i.e. a context effect). Thus, even though previous Australian studies find lower survival times in geographically remote and socioeconomically disadvantaged areas, this does not mean that areas per se are important in terms of influencing the survival probability of the area's residents. Ecologic studies leave open the possibility that area variation in survival is an artefact of varying population compositions, and unless these are taken into account (which aggregate studies cannot do), area- and individual-level sources of variation remain confounded. It therefore remains an unanswered question as to whether geographically remote and socioeconomically disadvantaged areas are important determinants of colorectal cancer survival in Australia.

### Survival analyses

The survival analysis will focus on cause-specific survival for all colorectal cancer patients diagnosed in Queensland between 1996 and 2005, with a minimum follow-up of two years to 31^st ^December 2007. Cause-specific survival will be used, since relative survival has been suggested to under-estimate social inequalities [48]. The QCR, like most other Australian registries, codes the cause of death using information from the ABS mortality file, pathology laboratories and other cancer-specific data sources, increasing the accuracy in the cause of death codes. Patients who died of causes other than CRC, or were still alive as at 31^st ^December 2007 will be censored. Survival time will be measured as the number of days from date of diagnosis to date of death, or the censoring date, whichever comes first.

### Multilevel models

The multilevel analyses will be conducted using a multilevel Cox proportional hazards model [49] with Level 1 comprising the individual-level factors (such as risk factors) and level 2 the indicators of geographic remoteness and area socioeconomic disadvantage. This approach extends traditional (single-level) proportional hazards modelling by incorporating a random intercept that reflects the average survival outcome (time or probability) for each area. The analyses will be undertaken in Mlwin 2.15 with the Cox survival macro, and the models will be estimated using the iterative generalised least squares algorithm (Poisson first order marginal quasi-likelihood).

The study's objectives will be addressed using a four-stage modelling strategy:

**Model 1**: (Objective 1): First, a null (intercept only) model will be specified to quantify the extent of area-level variation (i.e. variation between SLA) in colorectal cancer survival

**Model 2**: (Objective 2): Extends Model 1 by including individual-level factors (patient characteristics, disease stage, co-morbidity, and access to health care and treatment services) as fixed effects. This will tell us how much of the area-variation in colorectal cancer survival is due to these compositional factors; and also assess the contribution of each individual-level factor to survival.

**Model 3**: (Objective 3): Extends Model 2 by including the measures of geographic remoteness and area socioeconomic disadvantage as fixed effects. This will show us how much of the area-variation in colorectal cancer survival is due to these factors (independent of individual-level factors). This model will also assess the associations between geographic remoteness, area disadvantage and survival.

**Model 4**: (Objective 4): In this model we specify cross-level interactions between the individual-level factors and geographic remoteness and area disadvantage. This analysis will tell us if the relationship between colorectal cancer survival and each of the individual-level factors differs as a function of geographic remoteness or area disadvantage. For example, if the association between occupation and colorectal cancer survival is the same in both geographically remote and accessible areas, and affluent and deprived areas.

## Discussion

Colorectal cancer is the most commonly diagnosed cancer in Australia. The health inequalities for rural and disadvantaged populations are often documented but continue to widen. Our capacity to intervene is currently limited because very little is known about the reasons for the inequalities in colorectal cancer survival. The results from this study will inform our understanding of the causes of inequalities in colorectal cancer survival that can be addressed through improvements in health policy and practice aimed at improving population health and reducing inequalities that result from geographic remoteness and area disadvantage.

To our knowledge this study will be the first of its kind in Australia to investigate key area-level and individual-level components of colorectal cancer survival after adjusting for spread of disease, using a multilevel analytical approach on a combination of routine population-based data, longitudinal research data and original data on cancer stage, and applying emerging statistical and spatial technology.

## Competing interests

The authors declare that they have no competing interests.

## Authors' contributions

PB led the writing for this manuscript and wrote the introduction, measures, and discussion. GT wrote sections relating to the multilevel modelling components all authors contributed to the design and data collection protocols, and wrote sections of the grant application that was used in the paper. All authors read and approved the manuscript.

## Pre-publication history

The pre-publication history for this paper can be accessed here:

http://www.biomedcentral.com/1471-2407/10/24/prepub
